# A Hypoxia-Related lncRNA Signature Correlates with Survival and Tumor Microenvironment in Colorectal Cancer

**DOI:** 10.1155/2022/9935705

**Published:** 2022-07-08

**Authors:** Xinyang Zhong, Xuefeng He, Yaxian Wang, Zijuan Hu, Deshui Yu, Huixia Huang, Senlin Zhao, Ping Wei, Dawei Li

**Affiliations:** ^1^Department of Colorectal Surgery, Fudan University Shanghai Cancer Center, Shanghai, China; ^2^Department of Oncology, Shanghai Medical College Fudan University, Shanghai, China; ^3^Department of Pathology, Fudan University Shanghai Cancer Center, Shanghai, China; ^4^Cancer Institute, Fudan University Shanghai Cancer Center, Shanghai, China; ^5^Institute of Pathology, Fudan University, Shanghai, China; ^6^Department of Gastrointestinal Oncology, Fifth People's Hospital of Shenyang, Shenyang, China

## Abstract

The hypoxic tumor microenvironment and long noncoding RNAs (lncRNAs) are pivotal in cancer progression and correlate with the survival outcome of patients. However, the role of hypoxia-related lncRNAs (HRLs) in colorectal cancer (CRC) development remains largely unknown. Herein, we developed a hypoxia-related lncRNA signature to predict patients' survival and immune infiltration. The RNA-sequencing data of 500 CRC patients were obtained from The Cancer Genome Atlas (TCGA) dataset, and HRLs were selected using Pearson's analysis. Next, the Cox regression analysis was applied to construct a risk signature consisting of 9 HRLs. This signature could predict the overall survival (OS) of CRC patients with high accuracy in training, validation, and entire cohort. This signature was an independent risk factor and exerted predictive ability in different subgroups. Functional analysis revealed different molecular features between high- and low-risk groups. A series of drugs including cisplatin showed different sensitivities between the two groups. The expression pattern of immune checkpoints was also distinct between the two clusters in this model. Furthermore, the high-risk group had higher immune, stromal, and ESTIMATE score and a more repressive immune microenvironment than the low-risk group. Moreover, MYOSLID, one of the lncRNAs in this signature, could significantly regulate the proliferation, invasion, and metastasis of CRC.

## 1. Introduction

According to the latest epidemiological statistics, colorectal cancer (CRC) is an important factor that threatens the survival of mankind [1]. In 2022, it has been predicted that CRC will still be the third most common tumor and the second leading cause of cancer-related death in the USA [1]. Although a high incidence and mortality rate exist in CRC, effective treatments are still limited due to the complexity of tumor biology and the tumor heterogeneity [2]. Tumor microenvironment (TME) is a complex and heterogenous population that is composed of cancer cells, stromal cells such as cancer-associated fibroblasts, immune cells, cytokine networks, and oxygen [3]. Hypoxia is a common event in large amounts of solid tumors due to high consumption of oxygen and disordered intratumor blood vessels [4, 5]. Thus, cancer cells undergo metabolic reprogramming towards a more glycolytic phenotype and adjust to such a hypoxic environment, also known as the Warburg effect [6]. Enhanced glycolytic activity in cancer cells helps them obtain enough intermediate to synthesize biomacromolecules and promotes lactate secretion, which leads to dysregulation of extracellular pH [4, 6]. Hypoxia-induced pathophysiological processes could help cancer cells gain a survival advantage over other cells which leads to an immunosuppressive tumor microenvironment, an unsatisfactory chemotherapeutic effect, and poor patient survival [4, 7].

Long noncoding RNA (lncRNA) is widely involved in cancer progression but has no potential to code for protein [8]. Emerging studies have proved that extracellular oxygen levels regulate the expression of lncRNA [9]. Moreover, the expression pattern of lncRNAs varies under hypoxia and normoxia [9]. Huan et al. observed that the expression of lncRNA LUCAT1 in CRC was upregulated under hypoxia, which can promote the growth and drug resistance of CRC [10]. In addition, it has been demonstrated that dysregulated lncRNA levels in cancer can regulate some key regulators related to hypoxia such as HIF1A [11]. For example, lncRNA SNHG11 is upregulated and correlated with poor prognosis in CRC patients. Mechanically, SNHG11 can bind to pVHL and protect HIF1A from the degradation, accumulating HIF1A in the cancer cells [12]. Moreover, the accumulation of HIF1A is essential for tumor metastasis because it is involved in a lot of oncogenic processes such as epithelial-mesenchymal transition (EMT) and metabolic reprogramming [13]. Therefore, it has been established that hypoxia and lncRNA could regulate each other and form a complex network which has a strong impact on a series of cancer hallmarks [11].

Both lncRNAs and hypoxic TME contribute to tumor malignancy. Hence, discerning a hypoxia-related lncRNA (HRL) panel and establishing a relevant model could evaluate patient's clinical prognosis [14]. In addition, it has been revealed that targeting certain HRL can be an effective method in cancer treatment [10, 12]. Recently, several studies have explored the possibility of developing a HRL signature to predict patients' survival and tumor microenvironment in multiple cancer types [14–16]. Nevertheless, application of the HRL signature in CRC is still unknown. Therefore, this research constructed a HRL signature in CRC using The Cancer Genome Atlas (TCGA) database. This signature could not only offer prognostic value but also describe the TME features and estimate clinical treatments of each patient.

## 2. Methods and Materials

### 2.1. Data Acquisition and Processing

The expression matrix (FPKM) of CRC patients was downloaded from TCGA database (https://portal.gdc.cancer.gov/). Then, the FPKM value was transformed into the TPM value. The study included a total of 500 CRC patients with the following criteria: (1) overall survival (OS) time is more than 30 days. (2) Clinical data includes T, M, N, and AJCC stages, age, and gender. R package “caret” was applied to divide 500 CRC patients into training and validation group with a ratio of 7 : 3. Subsequently, 31 hypoxia-related genes were collected based on previous studies and listed in the Supplementary Table S1 [17, 18].

### 2.2. Identification of Hypoxia-Related lncRNAs

A total of 14048 RNAs were considered as lncRNA according to the annotation file from GENCODE (https://www.gencodegenes.org). Moreover, package “limma” was used to discover differently expressed lncRNAs (*P* < 0.05 and ∣logFC | >1) between tumorous and nontumorous samples. Further, differently expressed lncRNAs whose expression was correlated with the expression of at least 1 hypoxia-related gene (*P* < 0.01 and ∣*R* | >0.4) were regarded as HRLs.

### 2.3. Construction of the Hypoxia-Related lncRNA Model

Univariate Cox regression analysis was applied to select HRLs related to the overall survival (OS) of CRC patients (*P* < 0.05). Next, the associated lncRNAs were further selected by least absolute shrinkage and selection operator (LASSO) Cox regression analysis using the “glmnet” package. Finally, several lncRNAs and their relevant coefficients were confirmed which were applied to establish a HRL model. The risk score = coefficient (lncRNA1) × expression (lncRNA1) + coefficient (lncRNA2) × expression (lncRNA2) + ⋯+coefficient (*n*) × expression (*n*).

### 2.4. Prognostic Evaluation

The Kaplan-Meier survival analysis was applied to find out the survival difference between different subgroups. The sensitivity and specificity of HRL signature were calculated by ROC analysis. Further, univariate and multivariate Cox regression analyses were used to validate whether the HRL signature was an independent risk factor.

### 2.5. Nomogram

The 1-, 3-, 5-, and 7-year survival probabilities of CRC patients in the training and total cohort were predicted by nomogram. Nomogram construction included risk score, AJCC stage, gender, and age for accurate prediction. Moreover, the discrimination and accuracy of the nomograms were evaluated by concordance index (C-index) and calibration curves.

### 2.6. Functional Enrichment Analysis

The R package “limma” was used to select differently expressed genes between high- and low-score groups for Gene Ontology (GO) and Kyoto Encyclopedia of Genes and Genomes (KEGG) analyses. Subsequently, the gene symbols of these genes were converted into ENTREZ ID. The gene set “h.all.v7.4.entrez.gmt” was downloaded from the msigDB database (http://www.gsea-msigdb.org/gsea/msigdb/collections.jsp#H) for Gene Set Enrichment Analysis (GSEA) studies. Similarly, the gene symbols were converted into ENTREZ ID and sorted by descending log fold changes (logFC).

### 2.7. Drug Sensitivity and Immunotherapy Response Prediction

Using package “pRRophetic,” predicted drug sensitivity of CRC patients to certain drug was calculated [19]. By entering patient's RNA-sequencing data, this package could calculate relevant IC_50_ (half-maximal inhibitory concentration) value based on its built-in dataset [19]. To predict immunotherapy response, TIDE (Tumor Immune Dysfunction and Exclusion) algorithm was applied [20]. The predicted efficiencies of the immune checkpoint blockade (ICB) treatment of each CRC patient could be obtained after feeding their RNA-Seq data on the official website (http://tide.dfci.harvard.edu/).

### 2.8. Mutation Analysis

The copy number variation (CNV) data of the TCGA-COAD and TCGA-READ cohort was downloaded from TCGA database (https://portal.gdc.cancer.gov/). Further, to analyze the CNV data of high- and low-score groups, R package “maftools” was used [21]. The mutation landscape of both groups was visualized through waterfall plot. Tumor mutation burden (TMB) and differently mutated genes were further explored to reveal the intrinsic mechanism that leads to the biological difference between the two groups.

### 2.9. Immune Infiltration Evaluation

Immune, stromal, and ESTIMATE scores of CRC patients were calculated using ESTIMATE algorithm [22]. Further, the CIBSERSORT algorithm analyzed the 22 immune cells proportion of each sample by simply inputting TPM values of the sequencing data [23]. TIMER2.0 is a user-friendly web server that provides a rounded analysis of tumor-infiltrating immune cells in the TCGA cohorts [24]. The immune infiltration data including XCELL, TIMER, QUANTISEQ, MCPCOUNTER, and EPIC was downloaded from TIMER2.0 (http://timer.cistrome.org/). Also, the expression of immune checkpoints and cytokines was analyzed in two groups.

### 2.10. ssGESA

Single-sample gene set enrichment (ssGSEA) can calculate the score of certain cell or pathway at the level of a single sample [25]. A series of immune gene sets were retrieved from a previous study and are listed in Supplementary Table S2 [26]. Further, the scores of these gene sets were calculated using “GSVA” package.

### 2.11. *In Vitro* Experiments

To further explore the potential therapeutic targets in CRC, a series of functional experiments were performed and the detailed methods are introduced in Supplementary methods.

### 2.12. Statistical Analysis

R software performed the majority of the bioinformatic analysis. Student's *t*-test was used to compare the difference between the two groups of normally distributed variables. The Wilcoxon rank-sum test was applied for variables that were not normally distributed. The chi-square test could compare the differences between the two groups of categorical variables. The Pearson regression analysis revealed the correlation between the two variables. The two-sided *P* value of less than 0.05 was considered statistically significant.

## 3. Results

### 3.1. Construction of a Hypoxia-Related lncRNA Signature

**Figure 1:**
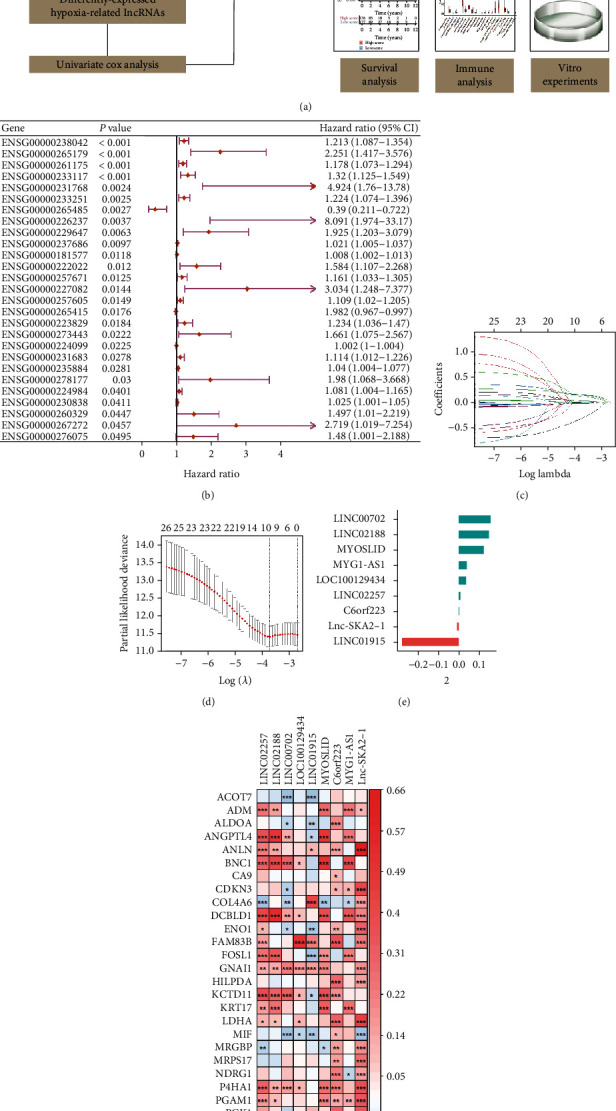
Construction of a hypoxia-related lncRNA signature. (a) Work flow chart of this study. (b) Forest plot of the 27 lncRNAs that correlated with the OS of CRC patients. (c and d) LASSO Cox regression analysis. (e) Coefficients of 9 lncRNAs. (f) Heat map that showed the relationship between hypoxia-related genes and 9 lncRNAs.

Figure 1(a) presented the overall design of the research. We obtained 159 differently expressed hypoxia-related lncRNAs between normal and tumorous samples. Among the 159 lncRNAs, 27 lncRNAs were correlated with the OS of CRC patients (Figure 1(b)). Package “caret” randomly separated CRC samples into a training (*n* = 353) and a validation (*n* = 147) cohort with a ratio of 7 : 3. There was no significant survival difference between the two cohorts (Figure S1A). We used training group to construct a HRL signature and test and entire cohort (*n* = 500) to verify the reliability of this model. LASSO regression analysis further removed 18 genes according to the value of lambda.min, and the remaining 9 genes with relevant regression coefficients were obtained to construct the prognostic model (Figures 1(c)–1(e)). As is shown in Figure 1(f), all of the 9 lncRNAs had positive correlations (*R* > 0.4) with at least one hypoxia-related gene. The expression of LINC02257, LINC02188, MYOSLID, C6orf223, MYG1-AS1, and Lnc-SKA2-1 was upregulated in tumor samples, while the expression of LINC00702, LOC100129434, and LINC01915 was downregulated in tumor samples (Figure S1B). The TPM values and coefficients of the 9 lncRNAs were applied to generate the risk score: risk score = TPM(LINC02257)∗0.01038406 + TPM(LINC02188)∗0.14814960 + TPM(LINC00702)∗0.15740894 + TPM(LOC100129434)∗0.03632290 − TPM(LINC01915)∗0.27678907 + TPM(MYOSLID)∗0.12183431 + TPM(C6orf223)∗0.00373216 + TPM(MYG1 − AS1)∗0.03923423 − TPM (Lnc − SKA2 − 1)∗0.00861392. According to the median value of each group, the patients were divided into high- and low-score group.

### 3.2. HRL Signature Correlated with Prognosis in CRC

**Figure 2:**
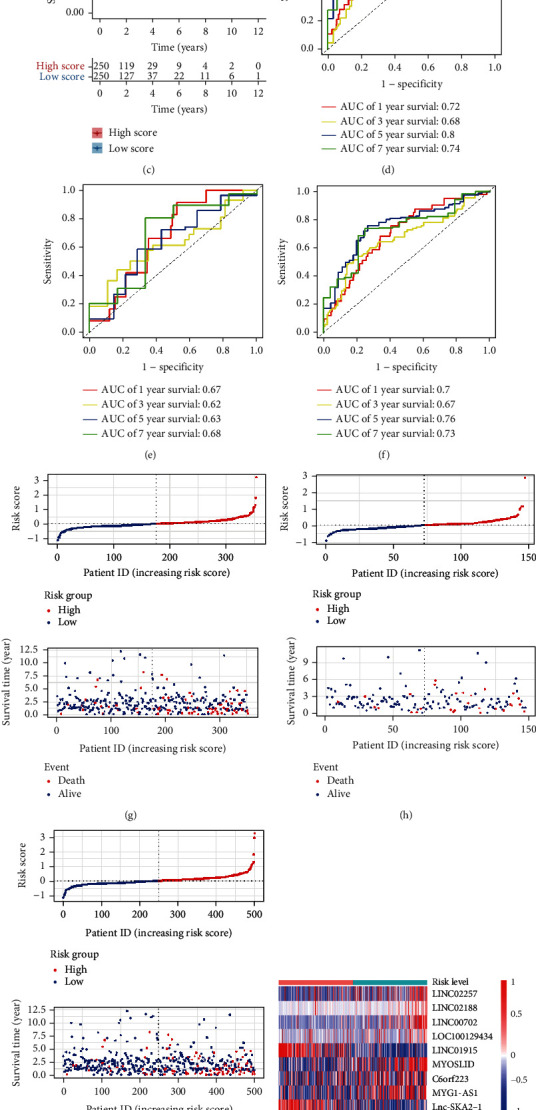
Predictive ability of the lncRNA signature. (a–c) The Kaplan-Meier plots in training, validation, and total cohort. (d–f) ROC curves in training, validation, and total cohort. (g–i) Risk curves and scatter plots in training, validation, and total cohort. (j–l) Heat map that showed the expression pattern of 9 lncRNAs in high- and low-risk group.

The Kaplan-Meier survival analysis showed that the high-score group exhibited a much shorter OS time than the low-score group in training, testing, and entire group (Figures 2(a)–2(c)). Then, the calculated the AUC value of 1-, 3-, 5-, and 7-year survival in all 3 cohorts confirmed the outstanding predictive ability of HRL model (Figures 2(d)–2(f)). The 5-year survival rate is commonly regarded as an important indicator to evaluate the outcomes of tumor patients. The AUCs for 5-year OS prediction in training, validation, and entire cohort were 0.8, 0.63, and 0.76, respectively, providing an effective basis for clinicians to judge the prognosis of patients (Figures 2(d)–2(f)). The distribution of survival time, survival status, and risk score were visualized by risk curves and scatter plots (Figures 2(g)–2(i)). In addition, the expression pattern of the 9 HRLs between high- and low-score groups was also different (Figures 2(j)–2(l)). Overall, the results demonstrated that the HRL signature exerts outstanding prognostic ability in CRC patients.

### 3.3. Clinical Performance of the lncRNA Signature

**Figure 3:**
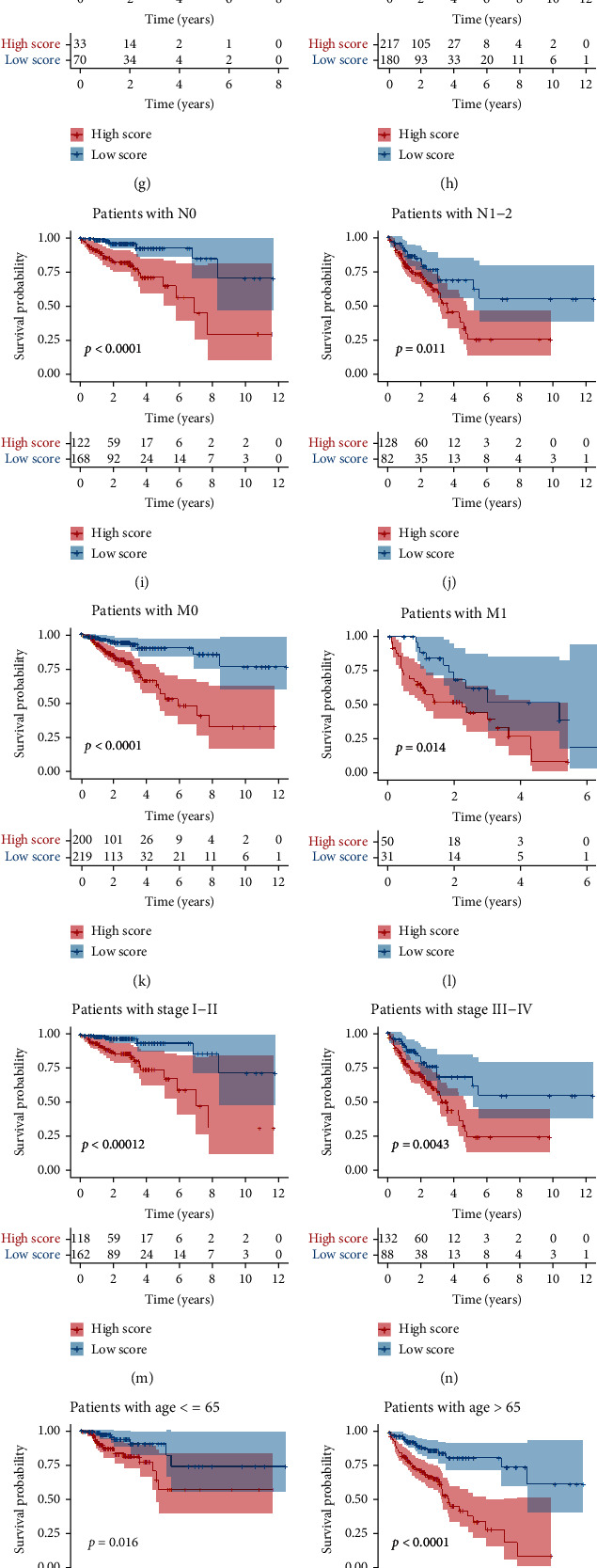
Predictive ability of the lncRNA signature. (a–c) Forest plot of the univariate Cox regression in training, validation, and total cohort. (d–f) Forest plot of the multivariate Cox regression in training, validation, and total cohort. (g–r) The Kaplan-Meier plots in different subgroups.

Univariate Cox regression analysis revealed the *P* values of lncRNA signature, age, and AJCC stage to be less than 0.05, indicating their association with patients' OS (Figures 3(a)–3(c)). Multivariate Cox regression analysis discovered that the *P* values of HRL signature were all less than 0.01 and the values of HR were all more than 2, demonstrating that the HRL signature is an independent risk factor in all three cohorts (*P* < 0.05) (Figures 3(d)–3(f)). The clinicopathological characteristics of both the training and validation sets were summarized in Table 1. We aimed to find out whether the HRL signature showed predictive abilities in different subgroups. Interestingly, the lncRNA signature could still exert predictive function in the majority of the subgroups (11 out of 12) (Figures 3(g)–3(r)). The relationship among clinical factors, gene expression, and risk level were shown using a heat map (Figure S2A). Moreover, the risk score in T3-4, N1-2, M1, and III-IV stage was significantly higher than in T1-2, N0, M0, and stages I-II (Figure S2B).

### 3.4. Construction of a Nomogram

**Figure 4:**
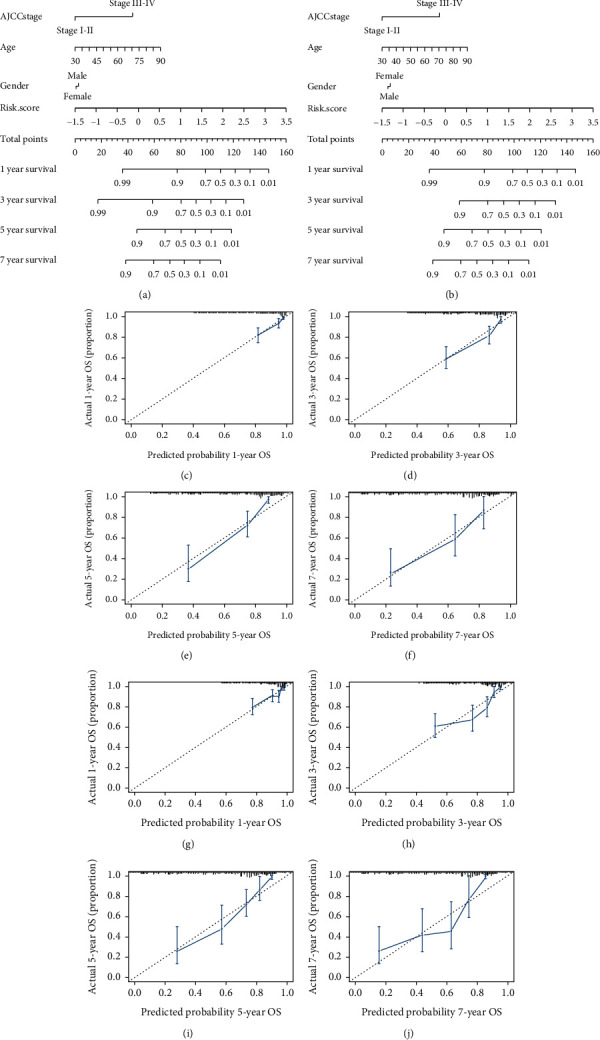
Nomogram. (a and b) Nomogram of training and total cohort. (c–j) The 1-, 3-, 5-, and 7-year calibration curves in training and total cohort.

Risk score, AJCC stage, gender, and age were used to construct the nomograms for predicting the 1-, 3-, 5-, and 7-year OS in both training cohort and entire cohort (Figures 4(a) and 4(b)). In the study, the patients were given a nomogram-based risk score and were estimated for survival probability. In addition, 1-, 3-, 5-, and 7-year calibration curves were drawn for both cohorts, which indicated that the nomogram showed high accuracy and stability (Figures 4(c)–4(j)). The concordance index of the nomogram in training and entire cohort was 0.78 and 0.76, respectively.

### 3.5. Gene Enrichment Analysis

**Figure 5:**
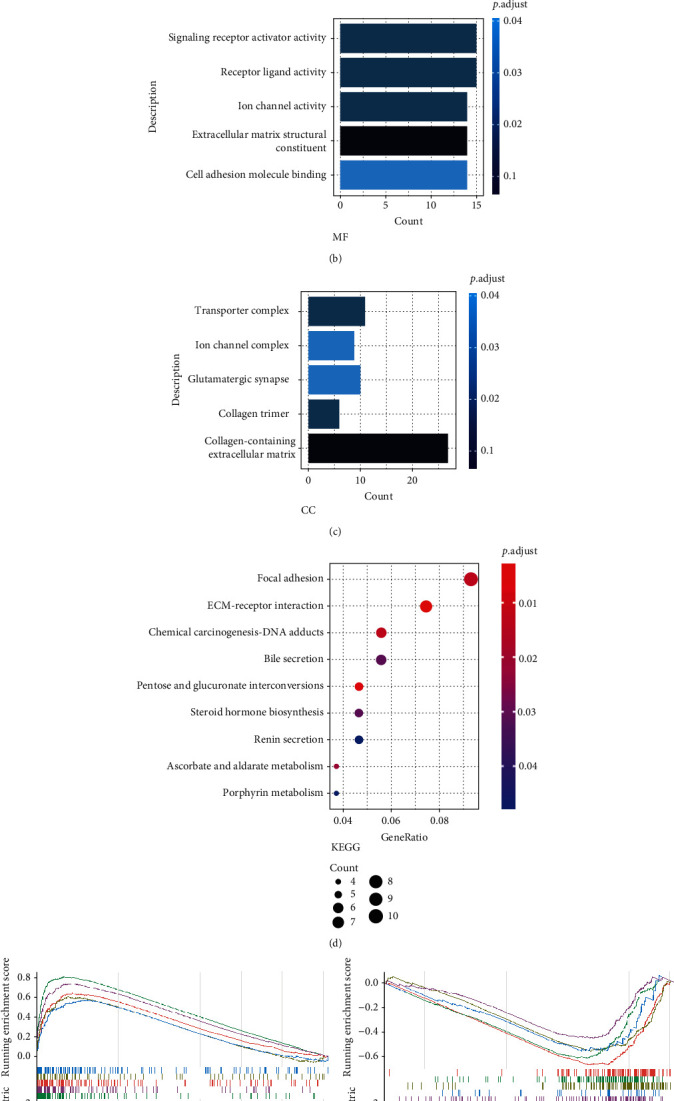
Functional analysis. (a–c) Bar plots that include representative GO terms in biological process (a), molecular function (b), and cellular component (c). (d) Bubble plot that describes KEGG terms. (e and f) Enrichment plots that showed enriched pathways in high- (e) and low-risk groups (f).

A series of functional analyses including GO, KEGG, and GESA were performed to determine the potential pathway or process that leads to the different clinical outcomes between the two groups. The R package “limma” was applied to select differently expressed genes between high- and low-risk subgroups, and then, these genes were subjected to GO and KEGG analyses. Representative GO terms included biological process (BP), molecular function (MF), and cellular component (CC). The BP included positive regulation of cytosolic calcium ion concentration, extracellular structure organization, extracellular matrix organization, calcium ion transport, and BMP signaling pathway (Figure 5(a)); the MF included signaling receptor activator activity, receptor ligand activity, ion channel activity, extracellular matrix structural constituent, and cell adhesion molecule binding (Figure 5(b)); and the CC included transporter complex, ion channel complex, glutamatergic synapse, collagen trimer, and collagen-containing extracellular matrix (Figure 5(c)). Moreover, the KEGG results indicated that different signaling such as ECM-receptor interaction, pentose and glucuronate interconversions, chemical carcinogenesis-DNA adducts, focal adhesion, and bile secretion might be involved in the different prognosis between the two groups (Figure 5(d)). Gene sets from “h.all.v7.4.entrez.gmt” were used to analyze their activities in each group. Next, the 5 most enriched pathways in each group were selected after setting *P* < 0.05, *q* values <0.25, and abs (NES) > 1.5 as the criteria to judge the enriched pathways. As is shown in Figures 5(e) and 5(f), pathways such as HALLMARK_EPITHELIAL_MESENCHYMAL_TRANSITION and HALLMARK_HYPOXIA were enriched in the high-risk group, while HALLMARK_OXIDATIVE_PHOSPHORYLATION and HALLMARK_MYC_TARGETS were enriched in the low-risk group.

### 3.6. HRL Signature Correlated with the Therapeutic Effects of Chemotherapy and Immunotherapy

**Figure 6:**
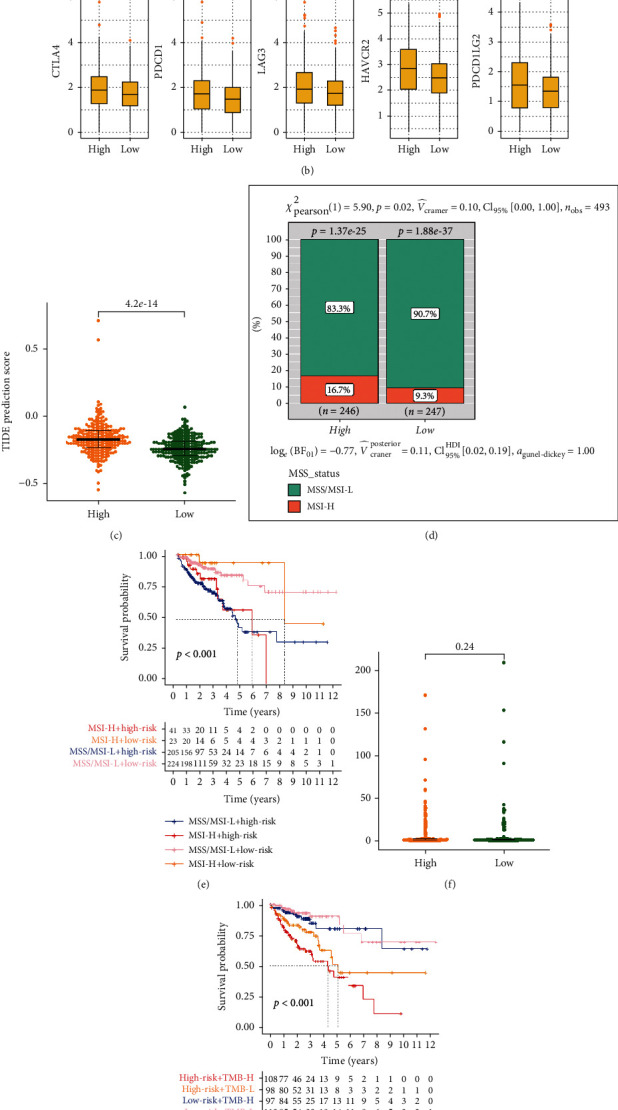
Treatment prediction. (a) Box plots that showed the IC_50_ of 6 drugs in high- and low-score group. (b) Box plots that showed the expression of immune checkpoints in high- and low-score group. (c) TIDE prediction score in high- and low-score group. (d) Bar chart that showed the proportion of MSS/MSI-L and MSI-H patients in high- and low-score group. (e) The Kaplan-Meier plots that compared the OS of MSS/MSI-L+high-risk, MSS/MSI-L+low-risk, MSI-H+high-risk, and MSI-H+low-risk group. (f) TMB levels of high- and low-score group. (e) The Kaplan-Meier plots that compared the OS of TMB-H+high-risk, TMB-H+low-risk, TMB-L+high-risk, and TMB-L+low-risk group.

The IC_50_ of several common drugs was calculated. We found the IC_50_ of masitinib, phenformin, and ruxolitinib was higher in the high-risk group, indicating that the high-risk group patients tend to be more resistant to these drugs (Figure 6(a)). On the contrary, the high-risk group might benefit from pazopanib, foretinib, and cisplatin, a widely used drug in CRC treatment (Figure 6(a)). Further, the mutation rate of the top 20 driver genes between two groups was visualized through waterfall plots (Figure S3A–B). A higher mutation rate of MUC16, NIPBL, PTPRK, MYO9A, LPA, TRRAP, APOB, and COL7A1 was found in the high-risk group (Figure S3C). Interestingly, the mutation rate of MUC16 in the high-risk group was 35% while in the low-risk group was 21% (Figure S3A–B). Moreover, the mutation of MUC16 in solid tumors has been reported to be correlated with a higher tumor mutational burden [27]. Immunotherapy is emerging as a promising method in cancer treatment. We discovered a significantly higher expression of CTLA4, PDCD1/PD1, LAG3, HAVCR2/TIM3, and PDCD1LG2/PD-L2 in the high-risk group (Figure 6(b)). Additionally, patients in the low-risk group showed a much lower TIDE score than its counterpart (*P* = 4.2e − 14), suggesting that immunotherapy had better therapeutic effects in these patients (Figure 6(c)). MSI status and TMB are two important hallmarks in predicting the efficiency of ICB therapy [28]. The results demonstrated that the high-risk group occupied a higher proportion of MSI-H patients (*P* = 0.02) (Figure 6(d)). What is more, the patients with MSI-L/MSS and low scores had better OS compared with those with MSI-L/MSS and high scores (Figure 6(e)). Similarly, the low-score MSI-H patients had better OS than the high score MSI-H patients (Figure 6(e)). However, the TMB analysis showed no significant difference between the two groups (Figure 6(f)), although the classification based on both TMB and risk score demonstrated that the risk score could predict patients' OS regardless of their TMB status (Figure 6(g)).

### 3.7. Hypoxia-Related lncRNA Signature Correlated with Immune Infiltration

**Figure 7:**
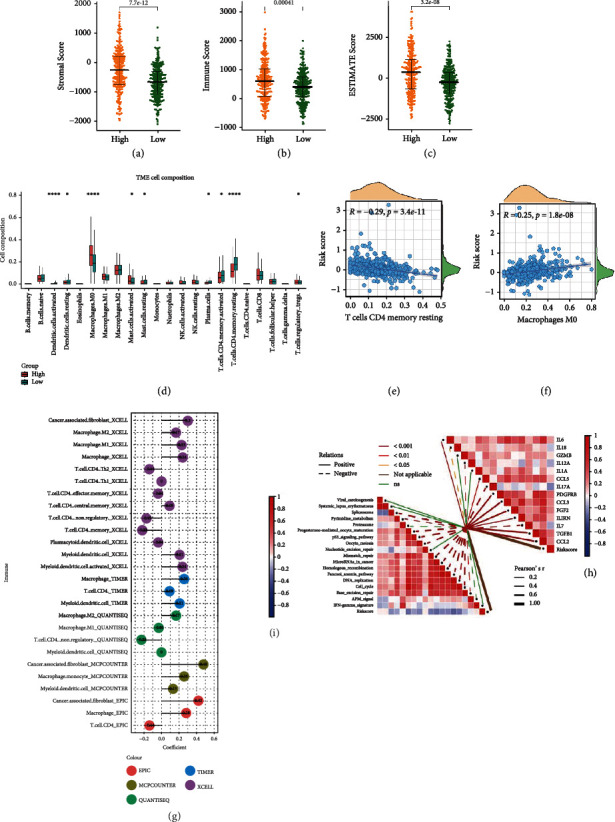
Tumor microenvironment estimation. (a–c) Beeswarm plots that compare the stromal score, immune score, and ESTIMATE score between high- and low-risk groups. (d) Box plots that showed the proportion of 22 immune cells in high- and low-score group. (e) Scatter plot that showed the correlation between T cell CD4 memory resting and risk score. (f) Scatter plot that showed the correlation between macrophage M0 and risk score. (g) Relationship between risk score and the proportion of stromal cells. (h) Correlation heat map that showed the relationship between risk score and the expression of cytokines. (i) Correlation heat map that showed the relationship between risk score and the activities of immunotherapy response-related pathways.

Exploring the infiltration pattern of immune cells could offer prognostic value and guide immunotherapy in CRC [28, 29]. Thus, a series of immune-related algorithms were applied to analyze the immune infiltration status of 500 CRC patients in the study. ESTIMATE algorithm revealed that the high-risk group had a higher stromal, immune, and ESTIMATE score (Figures 7(a)–7(c)). CIBERSORT results showed that the proportion of macrophages M0, mast cells activated, and T cells regulatory (Tregs) was higher, while the proportion of dendritic cells activated, dendritic cells resting, mast cells resting, plasma cells, T cells CD4 memory resting, and T cells CD4 memory activated was lower in the high-risk group (Figure 7(d)). Pearson analysis showed that the risk score had strong positive correlation with M0 macrophage and a strong negative correlation with T cells CD4 memory resting (Figures 7(e) and 7(f)). Further, the relationship between immune/stromal cells and HRL signature was explored using algorithms including XCELL, TIMER, QUANTISEQ, MCPCOUNTER, and EPIC. We observed that the relationship between macrophage, dendritic cell, CD4 T cell, and risk score in CIBERSORT was consistent with the majority of these algorithms (Figure 7(g)). Surprisingly, the risk score had strong positive correlation with cancer-associated fibroblasts (CAFs) (*R* = 0.3 in XCELL, *R* = 0.48 in MCPCOUNTER, and *R* = 0.42 in EPIC) (Figure 7(g)). Next, the expression patterns of cytokines were analyzed as they play an important role in CRC immunology. It was observed that most of the protumor cytokines had positive correlation with the risk score (Figure 7(h)). Scores of 18 immunotherapy response-related pathways were calculated by ssGESA. Surprisingly, an inverse correlation was observed between risk score and the scores of the pathways whose *P* value was less than 0.05 (Figure 7(i)).

### 3.8. MYOSLID Promotes the Progression of CRC

**Figure 8:**
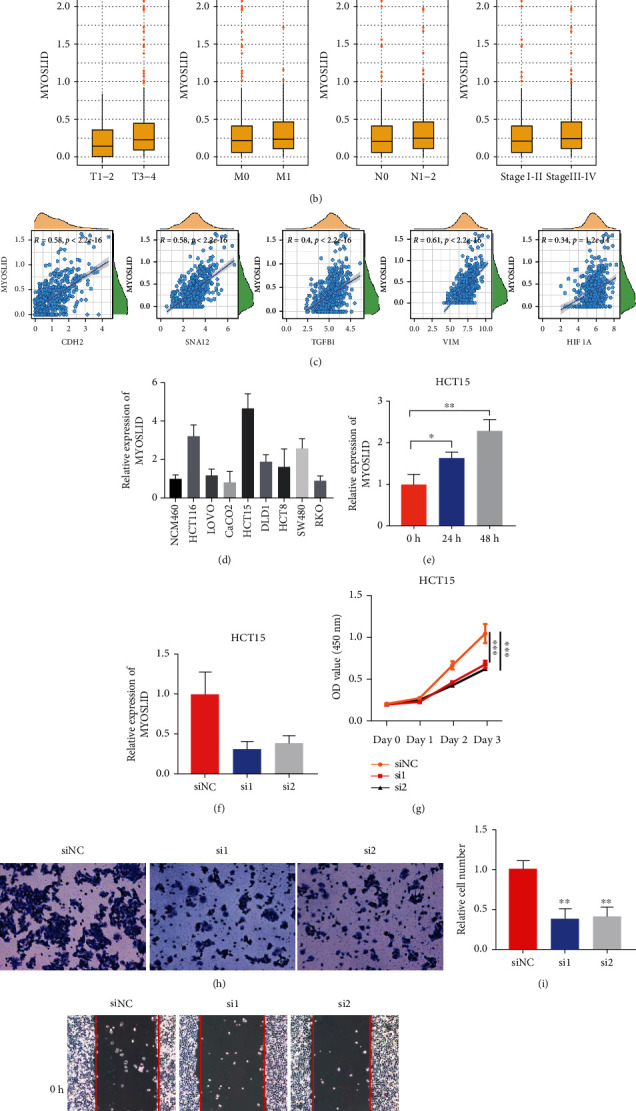
The role of MYOSLID in CRC. (a) The Kaplan-Meier plot that compared the OS between high- and low-MYOSLID groups. (b) Box plots that compared the expression of MYOSLID in subgroups with different clinical features. (c) Relationship between risk score and VIM, SNAI2, CDH2, TGFB1, and HIF1A. (d) The expression of MYOSLID (RNA level) in different cell lines. (e) The expression of MYOSLID under hypoxia. (f) The expression of MYOSLID (RNA level) before and after knocking down MYOSLID. (g) The OD450 value of HCT15 before and after knocking down MYOSLID. (h–k) Relative cell number in transwell assay. (i and j) Relative migration distance in wound healing assay.

There have been limited studies that discuss the oncogenic roles of MYOSLID. The previous research has indicated that MYOSLID was upregulated in the head and neck squamous cell carcinoma and contributed to tumor metastasis [30]. Similarly, MYOSLID could enhance the degree of malignancy by promoting the expression of MCL1 in gastric cancer [31]. However, no study has been reported that discusses the role of MYOSLID in CRC. We explored the relationship between patients' OS and expression of 9 HRLs and found that high expression of MYOSLID indicated a poor prognosis in TCGA CRC cohort (Figure 8(a) and Figure S4). Also, higher expression of MYOSLID can be found in the advanced T stage, N1-2 stage, and AJCC stage in TCGA CRC cohort (Figure 8(b)). In addition, our study found a strong positive correlation between MYOSLID and EMT-related genes including VIM, SNAI2, CDH2, and TGFB1 (Figure 8(c)). Similar correlation was also found between HIF1A and MYOSLID (Figure 8(c)). Therefore, we speculated that MYOSLID might also promote the progression of CRC. We examined the expression of MYOSLID in HCT116, HCT15, HCT8, DLD1, SW480, RKO, LOVO, CaCO2, and NCM460 and discovered that the expression of MYOSLID was highest in HCT15 (Figure 8(d)). Additionally, hypoxic environment can induce MYOSLID expression (Figure 8(e)). Two siRNAs were transfected into HCT15 to knock down MYOSLID (Figure 8(f)). It was observed that the knockdown of MYOSLID significantly reduced the proliferative ability of HCT15 (Figure 8(g)). Furthermore, results from the transwell assay and wound healing assay indicated that repressing MYOSLID expression could also inhibit invasion and migration in CRC (Figures 8(h)–8(k)). Therefore, we concluded that MYOSLID could function as an oncogene and could become a potential therapeutic target in CRC.

## 4. Discussion

Hypoxia has been regarded as a hallmark of the tumor microenvironment and exists in most of the solid tumors [4]. It has been studied that lack of oxygen could remodel tumor microenvironment by promoting abnormal angiogenesis and reducing extracellular pH levels and nutrient availability, resulting in immune suppression, drug resistance, and tumor progression [4, 32]. Herein, the 9 hypoxia-related lncRNAs were applied to construct a HRL signature. The HRL signature exhibited excellent predictive ability in predicting patients' OS. Additionally, the signature divided patients into two clusters, which showed distinctively different immune checkpoint expression and immune cell infiltration patterns. Finally, we experimentally validated the oncogenic function of MYOSLID in CRC.

The past decade has widely studied the function of lncRNA in CRC development, and the application of lncRNA as a prognostic model has been reported in a number of studies [33]. It has been demonstrated that hypoxia could induce the aberrant expression of lncRNAs, such as H19, HOTAIR, and NEAT1 to regulate tumor biology [34]. Moreover, lncRNA could upregulate the expression of HIF by increasing HIF-1*α* expression at both transcription and posttranscription levels [35]. Recently, a series of studies revealed that several hypoxia-related lncRNA signatures could provide predictive value [14, 16, 36]. In bladder cancer, a lncRNA signature consisting of 4 hypoxia-related lncRNAs was constructed and was found to exhibit excellent predictive performance [16]. Hence, it is pivotal to establish a HRL signature in CRC. Using bioinformatic analyses, we obtained 9 HRLs which included LINC02257, LINC02188, MYOSLID, C6orf223, MYG1-AS1, Lnc-SKA2-1, LINC00702, LOC100129434, and LINC01915 for further analysis. A HRL signature was established to calculate the risk scores of each patient. The Kaplan-Meier analysis and ROC curve revealed that the HRL signature showed potent accuracy in survival prediction. The signature was also an independent risk factor and could predict the OS in different subgroups. Hence, this novel hypoxia-related lncRNA signature could offer predictive value in CRC.

In CRC, chemotherapy is still the mainstay of treatment other than surgery [2]. Surprisingly, patients in the high-risk group were predicted to be more sensitive to cisplatin, a widely applied chemotherapy drug in CRC. Immune checkpoint therapy can offer lasting therapeutic effects and successfully improves the OS of patients [37, 38]. Nevertheless, the vast majority of CRC patients showed resistance to immune therapy, which greatly restricts their application in the clinic [39]. It has been demonstrated that TMB, mismatch repair deficiency, expression of immune checkpoint, and tumor-infiltrating immune cells are predictive biomarkers for therapeutic efficiency of ICB [28]. We found that patients in the high-score group had high mRNA level of CTLA4, LAG3, PDCD1, HAVCR2, and PDCD1LG2. Further, a larger population of MSI-H patients can be found in the high-score group. However, the TIDE algorithm predicted that patients in the low-score group can receive more benefits from ICB, which might be the result of a higher proportion of antitumor immune cell population in the low-score group.

Emerging evidence has demonstrated that the development and prognosis of CRC are tightly closed to TME. The proportion of a series of antitumor immune cells in CRC was significantly lower than in normal colorectal mucosa [29]. In addition, it has been observed that the suppressive myeloid cells were dominant in immunosuppressive CRC TME [29]. Studies have revealed that hypoxia could regulate the biological function of nearby stromal cells to shape an immunosuppressive microenvironment [4]. HIF-1 signaling has been reported to help cancer cells escape immune attack by inducing the expression of PD-L1 [40]. Hypoxia could also change the function of myeloid-derived suppressor cells (MDSCs) and promote their differentiation toward tumor-associated macrophages in the TME [41]. CAFs also facilitate the formation of an immunosuppressive TME [42]. The expression of several immunosuppressive cytokines including IL6, IL10, and TGF-*β* that are secreted by CAFs was also seen to be upregulated in hypoxia, inhibiting T cell-mediated cytotoxicity to suppress immune activation [43]. Our results revealed that a higher stromal score existed in the high-risk group, which could be a result of the enrichment of CAFs. Additionally, the HRL score was positively correlated with the proportion of CAF in XCELL, MCPCOUNTER, and EPIC. In our study, the HRL signature divided CRC patients into two clusters with distinct immune infiltration patterns. The result that the high-risk score group had a high infiltration of immune cells seemed to be perplexing. In fact, upregulation of immunosuppressive immune cells could contribute to the progression of cancer and indicates poor survival outcomes. Our study found a higher proportion of macrophages M0 and T cells regulatory (Tregs) in the high-risk group and a higher proportion of dendritic cells activated, dendritic cells resting, mast cells resting, plasma cells, T cells CD4 memory resting, and T cells CD4 memory activated in the low-risk group. Furthermore, the HRL score was negatively correlated with immunotherapy response-related pathways and was positively correlated with several protumor cytokines, which partly explained the poor response of the high-risk group to ICB therapy in TIDE algorithm.

It has been observed that lncRNAs selected using bioinformatic analysis could be more likely to be potential therapeutic targets for cancer treatment [14]. LINC02257, an enhancer RNA with DUSP10 as the target gene, has been reported to be upregulated and associated with the survival outcomes in multiple kinds of cancers [44]. MYG1-AS1 was regarded as a hypoxia-related lncRNA in hepatocellular carcinoma [45]. The role of LINC00702 in tumor development has been studied in several researches. LINC00702 functions as a tumor-suppressor gene by promoting PTEN expression in colorectal cancer and nonsmall cell lung cancer (NSCLC) while as an oncogene in malignant meningioma and ovarian cancer [46–49]. LINC01915 has been reported to inhibit the formation of CAFs through miR-92a-3p/KLF4/CH25H axis, which was consistent with our analysis that LINC01915 was downregulated in CRC compared with normal tissues [50]. MYOSLID has been reported to be a hypoxia- and autophagy-related lncRNA in HNSCC [51, 52]. Furthermore, the role of MYOSLID in HNSCC and gastric cancer has been validated using a series of *in vitro* and *in vivo* experiments [30, 31]. Our research focused on exploring the function of MYOSLID in CRC. The knockdown effects of MYOSLID were studied by applying a series of functional assays. The results revealed that MYOSLID could promote the progression of CRC by enhancing proliferation, invasion, and migration of CRC cells. Hence, MYOSLID could be a potential target in CRC therapy.

## 5. Conclusion

In summary, we developed a HRL signature that could predict the survival outcomes of CRC patients. Additionally, the HRL signature was correlated with drug sensitivity, immune checkpoint expression, and immune infiltration of CRC. Finally, we observed that MYOSLID could affect the biological function of CRC. These indicated that hypoxia-related lncRNA signature could be a novel biomarker in CRC diagnosis and promising therapeutic target in cancer treatment.

## Figures and Tables

**Table 1:** The clinicopathological characteristics of both the training and validation sets.

	Training cohort (*n* = 353)	Risk score		*P* value	Validation cohort (*n* = 147)	Risk score		*P* value
		High (*n* = 176)	Low (*n* = 177)			High (*n* = 73)	Low (*n* = 74)	
Gender				0.12				0.04
Female	156	85	71		72	42	30	
Male	197	91	106		75	31	44	
Age				0.87				0.93
≤65	156	77	79		65	32	33	
>65	197	99	98		82	41	41	
AJCC stage				0.0002				0.28
Stage I-II	205	85	120		75	34	41	
Stage III-IV	148	91	57		72	39	33	
T stage				<0.0001				0.87
T1-2	72	18	54		31	15	16	
T3-4	281	158	123		116	58	58	
M stage				0.01				0.44
M0	291	136	155		128	62	66	
M1	62	40	22		19	11	8	
N stage				0.0002				0.16
N0	213	89	124		77	34	43	
N1-2	140	87	53		70	39	31	

## Data Availability

In this study, all the data can be found in the following: (1) https://www.cancergenome.nih.gov/, (2) http://www.gsea-msigdb.org/gsea/msigdb/, (3) http://timer.comp-genomics.org/, (4) https://www.gencodegenes.org, and (5) http://tide.dfci.harvard.edu/.

## Supplementary Materials

Supplementary Materials Supplementary Figure S1: (A) the Kaplan-Meier analysis of training cohort and validation cohort. (B) Heat map of expression matrix of 9 lncRNAs in tumor and normal samples. Supplementary Figure S2: (A) heat map that described the correlation among risk level, clinical features, and hypoxia-related lncRNAs. (B) Risk score comparison in different T, M, N, and AJCC stage. Supplementary Figure S3: (A and B) waterfall plots of the top 20 mutated genes in two groups. (C) Differentially mutated genes between high- and low-risk groups. Supplementary Figure S4: the Kaplan-Meier analysis of LINC02257, LINC02188, LINC00702, LOC100129434, LINC01915, C6orf223, MYG1-AS1, and Lnc-SKA2-1. Supplementary Table S1: 31 hypoxia-related genes. Supplementary Table S2: gene lists used in ssGSEA. Supplementary Table S3: sequences of siRNA and primers used in this study. Supplementary Methods: in vitro experiments in this study.
