# Supplementary material for: A Hypoxia-Related lncRNA Signature Correlates with Survival and Tumor Microenvironment in Colorectal Cancer
Source: J Immunol Res. 2022 Jul 8;2022:9935705. doi: 10.1155/2022/9935705 (PMC9286950; doi:10.1155/2022/9935705)
Supplement: Supplementary Materials — Supplementary Figure S1: (A) the Kaplan-Meier analysis of training cohort and validation cohort. (B) Heat map of expression matrix of 9 lncRNAs in tumor and normal samples. Supplementary Figure S2: (A) heat map that described the correlation among risk level, clinical features, and hypoxia-related lncRNAs. (B) Risk score comparison in different T, M, N, and AJCC stage. Supplementary Figure S3: (A and B) waterfall plots of the top 20 mutated genes in two groups. (C) Differentially mutated genes between high- and low-risk groups. Supplementary Figure S4: the Kaplan-Meier analysis of LINC02257, LINC02188, LINC00702, LOC100129434, LINC01915, C6orf223, MYG1-AS1, and Lnc-SKA2-1. Supplementary Table S1: 31 hypoxia-related genes. Supplementary Table S2: gene lists used in ssGSEA. Supplementary Table S3: sequences of siRNA and primers used in this study. Supplementary Methods: in vitro experiments in this study. [file 9935705.f1.zip › Supplementary figures (1).docx]

**Supplementary Figures**

**
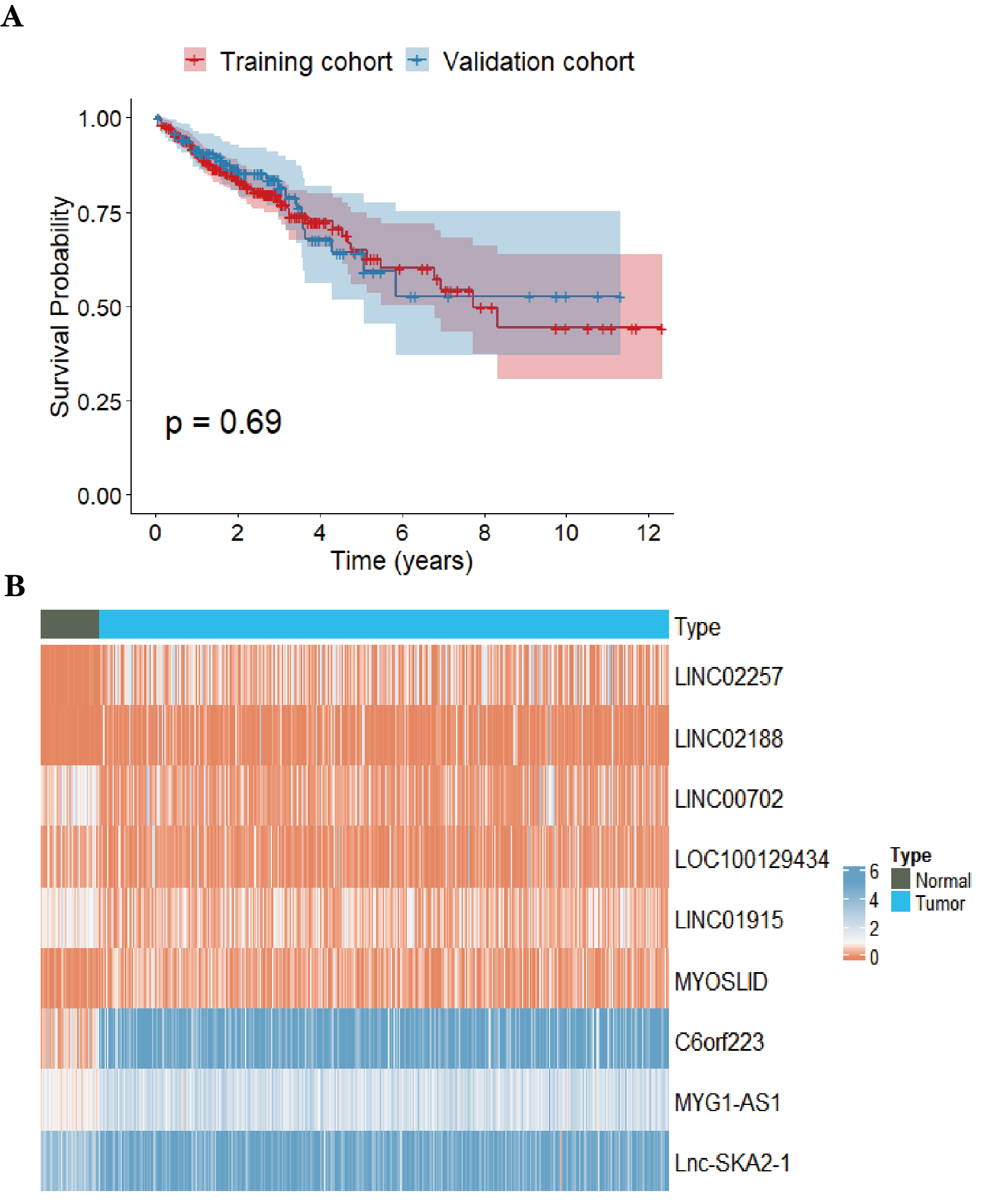
**

**Supplementary Figure S1|** (A) Kaplan–Meier plot that compared the OS between training and validation cohort. (B) Heatmap of expression matrix of 9 lncRNAs in tumor and normal samples.

**
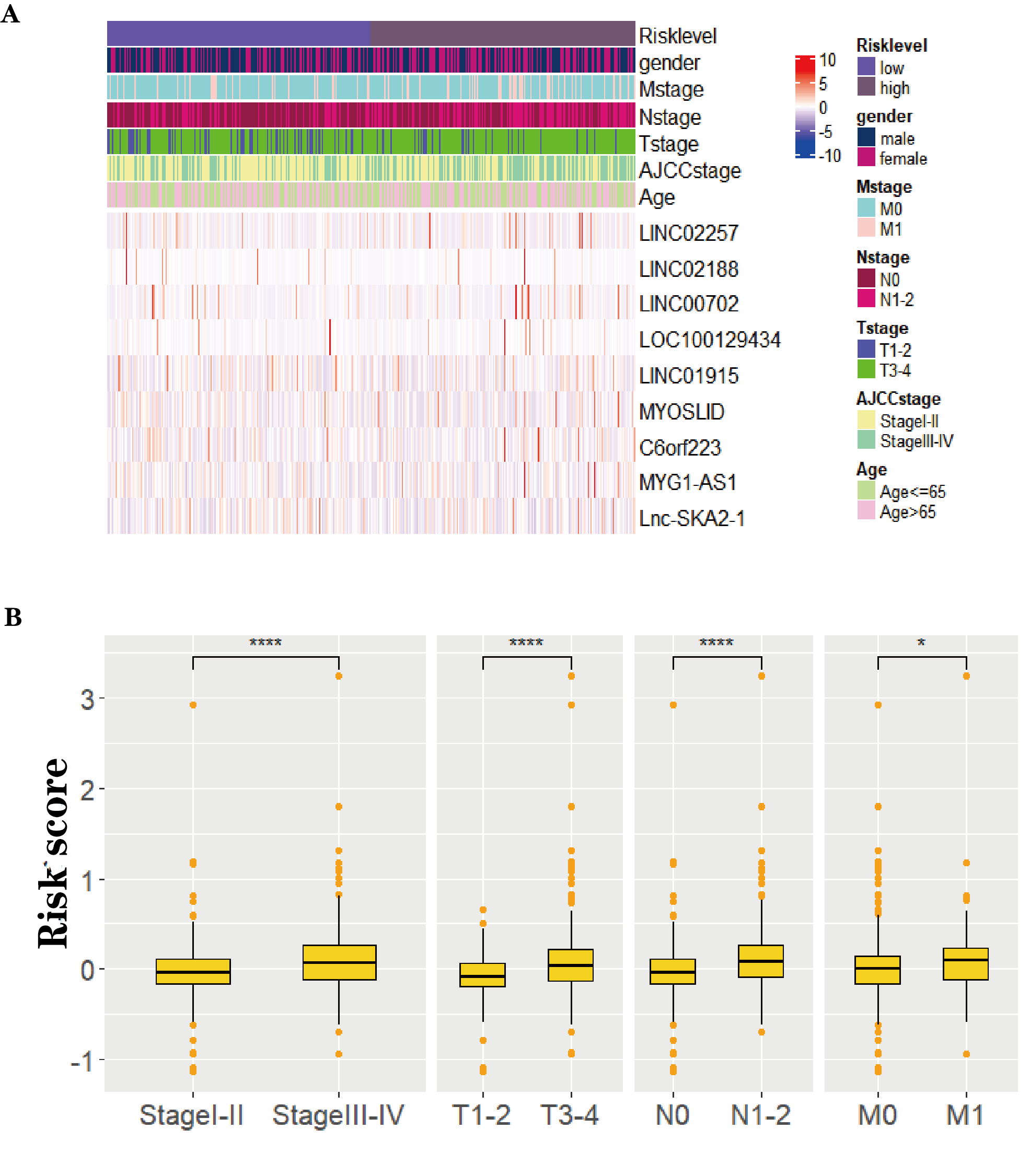
**

**Supplementary Figure S2|** (A) Heatmap that described the correlation among risk level, clinical features and hypoxia-related lncRNAs. (B) Box plots that showed the different levels of risk score in different T, M, N and AJCC stage.

**
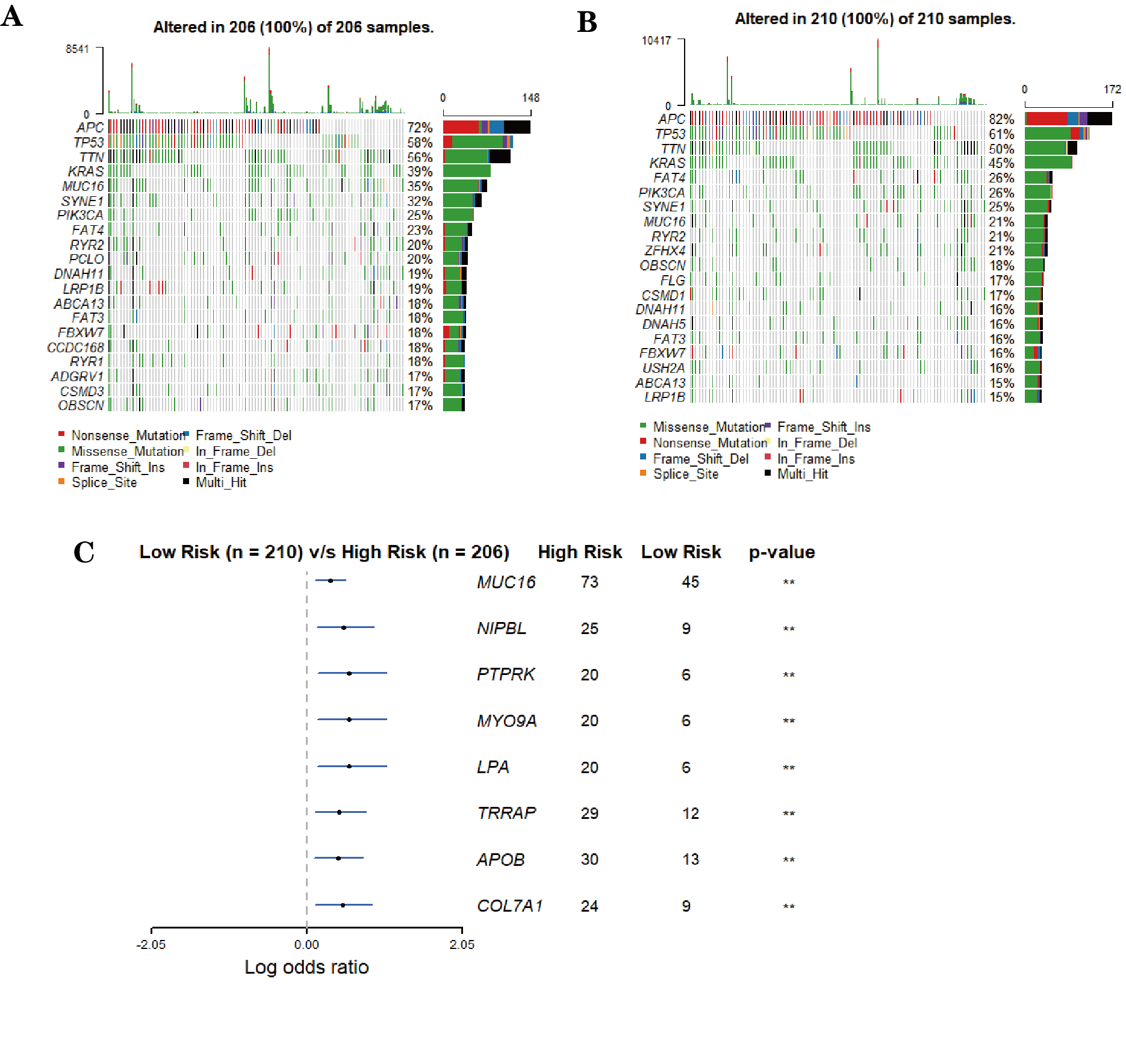
**

**Supplementary Figure S3|** (A-B) Waterfall plot of the top 20 mutated genes in two groups. (C) Differentially-mutated genes between high and low-risk group.

**
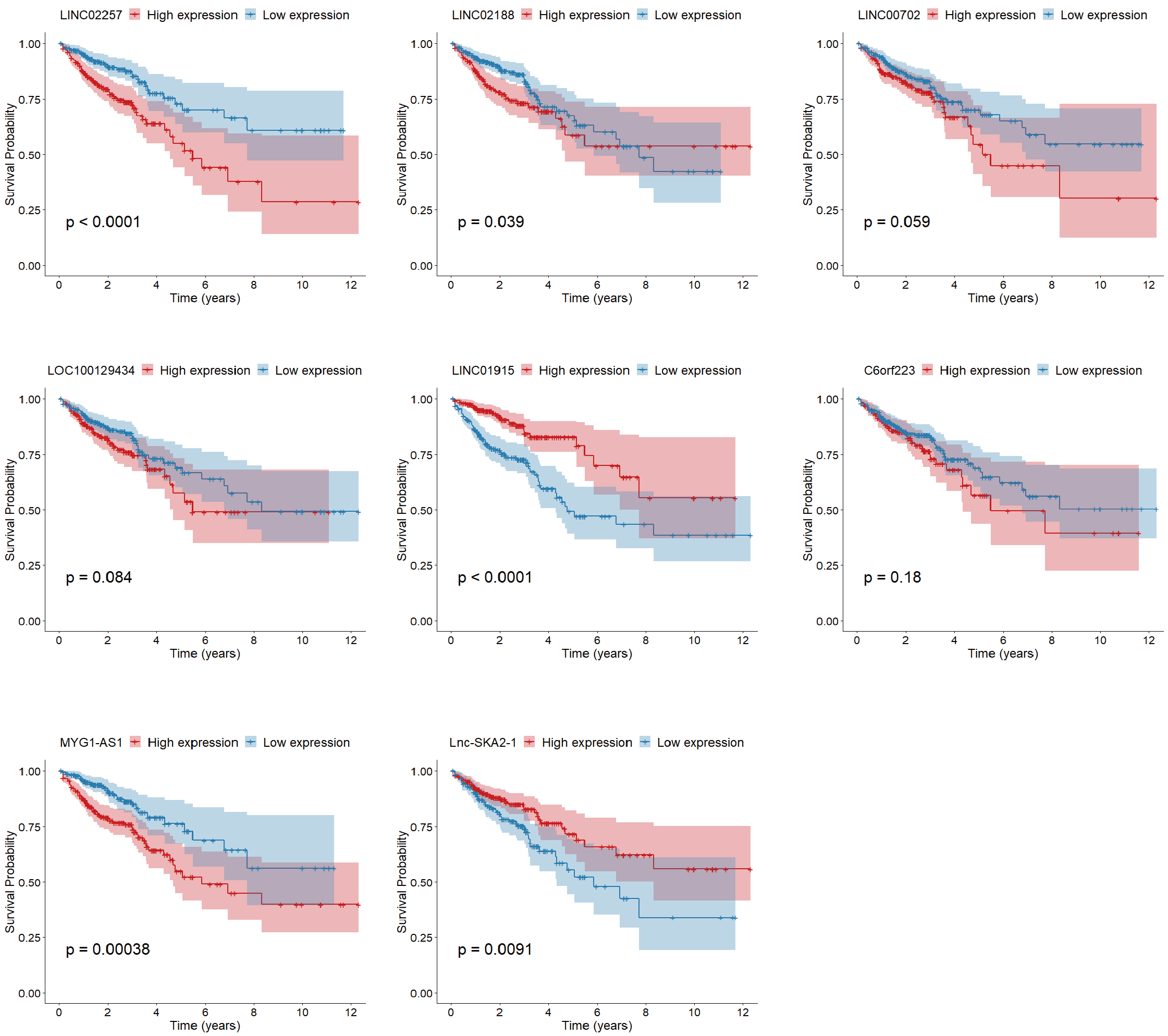
**

**Supplementary Figure S4|** Kaplan-Meier analysis of LINC02257, LINC02188, LINC00702, LOC100129434, LINC01915, C6orf223, MYG1-AS1 and Lnc-SKA2-1.
