# Supplementary material for: A Hypoxia-Related lncRNA Signature Correlates with Survival and Tumor Microenvironment in Colorectal Cancer
Source: J Immunol Res. 2022 Jul 8;2022:9935705. doi: 10.1155/2022/9935705 (PMC9286950; doi:10.1155/2022/9935705)
Supplement: Supplementary Materials — Supplementary Figure S1: (A) the Kaplan-Meier analysis of training cohort and validation cohort. (B) Heat map of expression matrix of 9 lncRNAs in tumor and normal samples. Supplementary Figure S2: (A) heat map that described the correlation among risk level, clinical features, and hypoxia-related lncRNAs. (B) Risk score comparison in different T, M, N, and AJCC stage. Supplementary Figure S3: (A and B) waterfall plots of the top 20 mutated genes in two groups. (C) Differentially mutated genes between high- and low-risk groups. Supplementary Figure S4: the Kaplan-Meier analysis of LINC02257, LINC02188, LINC00702, LOC100129434, LINC01915, C6orf223, MYG1-AS1, and Lnc-SKA2-1. Supplementary Table S1: 31 hypoxia-related genes. Supplementary Table S2: gene lists used in ssGSEA. Supplementary Table S3: sequences of siRNA and primers used in this study. Supplementary Methods: in vitro experiments in this study. [file 9935705.f1.zip › Supplementary methods and tables (1).docx]

**Supplementary methods**

**1. Cell Culture and siRNA Transfection**

Human CRC cell line HCT15 were obtained from Chinese Academy of Sciences Cell Bank, which was cultured in RPMI 1640 medium (Gibco, USA) that contains 1% penicillin/streptomycin (Gibco, USA) and 10% fetal bovine serum (Invitrogen, USA). MYOSLID siRNA#1, MYOSLID siRNA#2 and a negative control (Genomeditech, China) were transfected into HCT15 cells using RNAi MAX (Invitrogen). siRNAs targeting MYOSLID were listed in **Supplementary Table3**. For hypoxia treatment, CRC cell line HCT15 was cultured with 5% CO2, 1% O2 and 94% N2 for 24h and 48h.

**2. RNA Extraction and RT-qPCR**

Total RNA was extracted using TRIzol reagent (Beyotime Biotechnology, China), which was further quantified. Reverse transcription to cDNA was performed using ABScript II RT Mix for qPCR (ABclonal, China). TB Green® Premix Ex Taq™ II (TaKaRa, Japan) was used to perform Quantitative real-time PCR (qRT-PCR) and β-Actin was the reference gene. **Supplementary Table3** summarized all the primers in this study.

**3. Cell Counting Kit-8 Assay**

HCT15 cells at a density of 1000 cells per well were inoculated into 96-well plates and cultured in RPMI 1640 medium for 0, 1, 2, and 3 days. Then, CCK-8 reagent (Yeasen, China) was added into each well according to the manufacturer’s protocols and absorbance value at 450 nm was measured.

**4. Transwell Assay**

The lower chamber of transwell contained 0.6ml 1640 medium with 10% FBS. The upper chamber of transwell was precoated with Matrigel. A total of 40000 HCT15 cells which were suspended in FBS-free RPMI 1640 medium were added into the pore transwell inserts. After 48h, cells were fixed with 4% paraformaldehyde and then stained with crystal violet. Cell numbers were calculated under mircoscope.

**5. Cell Migration Assay**

HCT15 was grown in 6-well plates until they cover over 95% area of the well. In order to produce the wound, the tip of a 10μl pipettor was applied to scratch the cells. The wound was photographed under a 10x microscope at 0h and 24h. The migration ability was calculated using the formula: Migration ability= (Width at 0 h- Width at 24 h)/ Width at 24 h.

**Supplementary Table S1: Hypoxia-related Genes**

ACOT7

ADM

ALDOA

ANGPTL4

ANLN

BNC1

CA9

CDKN3

COL4A6

DCBLD1

ENO1

FAM83B

FOSL1

GNAI1

HILPDA

KCTD11

KRT17

LDHA

MIF

MRGBP

MRPS17

NDRG1

P4HA1

PGAM1

PGK1

SDC1

SLC16A1

SLC2A1

TPI1

TUBB6

VEGFA

**Supplementary Table S2: Immune Pathways and Immune Function Used in ssGSEA**

| **IFN-Gamma_signature** | TIGIT CD27 CD8A PDCD1LG2 LAG3 CD274 CXCR6 CMKLR1 NKG7 CCL5 PSMB10 IDO1 CXCL9 HLA-DQA1 CD276 STAT1 HLA-DRB1 HLA-E |
| --- | --- |
| **APM_signal** | B2M HLA-A HLA-B HLA-C TAP1 TAP2 |
| **Base_excision_repair** | FEN1 LIG1 NEIL3 PARP1 PARP2 PCNA POLE POLE2 UNG |
| **Cell_cycle** | BUB1 BUB1B CCNA2 CCNB2 CCNE1 CCNE2 CDC20 CDC25A CDC25C CDC6 CDK1 CDK2 CDKN2A DBF4 E2F1 E2F2 ESPL1 MAD2L1 MAD2L2 MCM2 MCM4 MCM6 MCM7 ORC1 ORC6 PCNA PLK1 SKP2 SMC3 TFDP1 TTK YWHAB |
| **DNA_replication** | DNA2 FEN1 LIG1 MCM2 MCM4 MCM6 MCM7 PCNA POLA2 POLE POLE2 PRIM1 PRIM2 RFC2 RFC3 RFC4 RFC5 RNASEH2A RPA1 RPA3 |
| **Fanconi_anemia_pathway** | BLM BRCA1 BRCA2 BRIP1 EME1 ERCC4 FANCA FANCB FANCD2 FANCI PALB2 RAD51 RAD51C RMI1 RMI2 RPA1 RPA3 TOP3A UBE2T |
| **Homologous_recombination** | BLM BRCA2 EME1 RAD51 RAD51C RAD54L RPA1 RPA3 TOP3A XRCC2 XRCC3 |
| **MicroRNAs_in_cancer** | BRCA1 CCNE1 CCNE2 CDC25A CDC25C CDCA5 CDKN2A DNMT1 E2F1 E2F2 EZH2 KIF23 STMN1 TRIM71 |
| **Mismatch_repair** | EXO1 LIG1 PCNA RFC2 RFC3 RFC4 RFC5 RPA1 RPA3 |
| **Nucleotide_excision_repair** | CETN2 ERCC4 LIG1 PCNA POLE POLE2 RFC2 RFC3 RFC4 RFC5 RPA1 RPA3 |
| **Oocyte_meiosis** | AURKA BUB1 CCNB2 CCNE1 CCNE2 CDC20 CDC25C CDK1 CDK2 ESPL1 FBXO5 MAD2L1 MAD2L2 PLK1 SGOL1 SMC3 YWHAB |
| **p53_signaling_pathway** | CCNB2 CCNE1 CCNE2 CDK1 CDK2 CDKN2A GTSE1 PPM1D RFWD2 RRM2 |
| **Progesterone-mediated_oocyte_maturation** | BUB1 CCNA2 CCNB2 CDC25A CDC25C CDK1 CDK2 MAD2L1 MAD2L2 PLK1 |
| **Proteasome** | IFNG PSMA4 PSMB2 PSMB4 PSMC4 PSMD4 PSMD7 |
| **Pyrimidine_metabolism** | CTPS1 DTYMK POLA2 POLE POLE2 PRIM1 PRIM2 RRM2 TYMS |
| **Spliceosome** | HNRNPM LSM3 LSM4 LSM5 MAGOHB PRPF19 SF3B2 SF3B3 SF3B4 SNRNP40 SNRPA1 SNRPC USP39 WBP11 |
| **Systemic_lupus_erythematosus** | H2AZ2 H2AZ1 H2AC4 H2AC11 H2AC12 H2AC13 H2AC17 H2BC4 H2BC5" H2BC7 H2BC11 H2BC12 H2BC13 H2BC15 H2BC17 H3C2 H3C4 H3C10 H4C1 H4C2 H2AC21 |
| **Viral_carcinogenesis** | CCNA2 CCNE1 CCNE2 CDC20 CDK1 CDK2 CDKN2A CREB3L4 GTF2E1 H2BC4 H2BC5 H2BC7 H2BC11 H2BC12 H2BC13 H2BC15 H2BC17 H4C1 H4C2 H2BC21 H2BC18 SKP2 YWHAB |

**Supplementary Table S3: Sequences of siRNA and Primers Used in This Study**

| **siRNA** |  |
| --- | --- |
| MYOSLID siRNA#1 | 5’-GCUCAAGUCAAACGCAUUUTTAAAUGCGUUUGA  CUUGAGCTT-3’ |
| MYOSLID siRNA#2 | 5’-GGACAUGGCUGAGCAUGUUTTAACAUGCUCAGC  CAUGUCCTT-3’ |
| **Primers** |  |
| MYOSLID -F | 5’-AAGAGGGAGTGGGAGTTAGGC-3’ |
| MYOSLID -R | 5’-CACTGTGGTGGGATCTGCAAG-3’ |
| β-Actin-F | 5’-ATCATGTTTGAGACCTTCAACA-3’ |
| β-Actin-R | 5’-CATCTCTTGCTCGAAGTCCA-3’ |
